# Associations of HbA_1c_ and educational level with risk of cardiovascular events in 32 871 drug-treated patients with Type 2 diabetes: a cohort study in primary care

**DOI:** 10.1111/dme.12145

**Published:** 2013-03-13

**Authors:** C J Östgren, J Sundström, B Svennblad, L Lohm, P M Nilsson, G Johansson

**Affiliations:** 1 Department of Medical and Health Sciences, Linköping UniversityLinköping; 2 Department of Medical Sciences, Uppsala UniversityUppsala; 3 Uppsala Clinical Research Center, Uppsala UniversityUppsala; 4 AstraZeneca NordicSödertälje; 5 Department of Clinical Sciences, Lund University, Malmö, University HospitalMalmö; 6 Department of Public Health and Caring Sciences, Uppsala UniversityUppsala, Sweden

## Abstract

**Aims:**

To explore the association of HbA_1c_ and educational level with risk of cardiovascular events and mortality in patients with Type 2 diabetes.

**Methods:**

A cohort of 32 871 patients with Type 2 diabetes aged 35 years and older identified by extracting data from electronic patient records for all patients who had a diagnosis of Type 2 diabetes and had glucose-lowering agents prescribed between 1999 and 2009 at 84 primary care centres in Sweden. Associations of mean HbA_1c_ levels and educational level with risks of cardiovascular events and all-cause mortality were analysed.

**Results:**

The associations of HbA_1c_ with risk of all-cause and cardiovascular mortality were J-shaped, with the lowest risk observed for cardiovascular mortality at an HbA_1c_ level of 51 mmol/mol (6.8%) for subjects on oral agents and 56 mmol/mol (7.3%) in insulin-treated patients. The lowest risk observed for all-cause mortality was at an HbA_1c_ level of 51 mmol/mol (6.8%) for subjects on oral agents and 56 mmol/mol (7.3%) in insulin-treated patients. There was an increased risk for cardiovascular death [hazard ratio 1.6 (1.2–2.1), *P* = 0.0008] at the lowest HbA_1c_ decile for subjects in the low education category. For subjects with higher education there was no evident J curve for cardiovascular death [hazard ratio 1.2 (0.8–1.6), *P* = 0.3873].

**Conclusions:**

Our results lend support to the recent American Diabetes Association/ European Association for the Study of Diabetes position statement that emphasizes the importance of additional factors, including the propensity for hypoglycaemia, which should influence HbA_1c_ targets and treatment choices for individual patients.

(Clinical Trials Registry No; NCT 01121315)

## Introduction

In Type 2 diabetes, improved glycaemic control reduces the risk of microvascular complications, whereas the role of intensive glycaemic control in reducing macrovascular complications is less clear 1,2. Benefits from early intervention to achieve glycaemic control are proven, but intensity of control has been debated 3. In 2008, the results from three major cardiovascular outcome studies of intensive glycaemic control in patients with Type 2 diabetes were presented 1,4,5. All three studies failed to show that achievement of intensified glycaemic control was associated with reduction of cardiovascular risk, and the Action to Control Cardiovascular Risk in Diabetes (ACCORD) study actually reported a 22% increase in total deaths in the intensively treated group 5, which led to the conclusion that the findings identified a previously unrecognized harm of intensive glucose-lowering in high-risk patients with Type 2 diabetes. The harm associated with severe hypoglycaemia might counterbalance the potential benefit of intensive glucose-lowering treatment 6–8. Since then some observational studies have reported a J- or U-shaped 9,10 association between HbA_1c_ levels and mortality where low and high levels of HbA_1c_ were linked with higher rates of death. This has been shown for subjects with Type 2 diabetes on a combination oral regimen with a sulphonylurea plus metformin and well as in subjects on insulin treatment 9 and in subjects who were aged ≥ 60 years 10. However, a recent Swedish observational study showed a progressively increased total mortality with increasing HbA_1c_ levels and no J-shaped risk curves 11.

Although not consistently, some previous results have suggested that there are important indicators of social deprivation, such as low education, which predict mortality over and above diabetic health status itself 12.

Our hypothesis was that the association between glycaemic control and cardiovascular events may differ in different groups of educational level. Thus, in this cohort study of patients with Type 2 diabetes in primary care, the primary objective was to clarify if a linear or J-shaped association exists between HbA_1c_ levels and cardiovascular morbidity and mortality. A second aim was to explore if such associations were dependent on socio-economic background in terms of educational achievement.

## Patients and methods

### Study sample

This observational study was based on patients with Type 2 diabetes in Swedish primary care based on the Retrospective Epidemiological Study to Investigate Outcome and Mortality with Glucose Lowering Drug Treatment in Primary Care (ROSE) study sample. For this study, data were extracted in 2010 from electronic patient records from 84 primary care centres in Sweden by the Pygargus Customized eXtraction Program 13. The primary care centres were chosen to provide a good representation of Swedish primary care. All data between the years 1999 and 2009 were extracted for all 58 326 patients with a diagnosis of Type 2 diabetes [International Classification of Diseases (ICD)-9 code 250, ICD-10 codes E10-E14] and/or prescription of drug within Anatomic Therapeutic Chemical classification system class A10. We excluded patients who were not subjected to pharmacological treatment for diabetes (*n* = 16 973), were under the age of 35 years (*n* = 876) and patients who had incomplete data on HbA_1c_ at baseline (*n* = 7606), rendering a final sample of 32 871 patients for further analyses.

### Baseline examinations

Baseline was defined as the first time a patient was diagnosed with Type 2 diabetes or was prescribed an anti-diabetic drug. All baseline values were calculated as the mean of the values in a period of 6 months before to 6 months after the index date. Time-varying variables were determined as annually updated means—the baseline value was used for the period from the index date to 1 year; for the period from year *i* to *i*+1, the value used will be the mean of all values between year *i*−1 and year *i*.

Patients' age and sex were determined using the unique personal identification number allocated to all Swedish citizens. Baseline data were extracted from electronic patient charts for the variables of systolic and diastolic blood pressure; total, LDL and HDL cholesterol; serum triglycerides; HbA_1c_ values; lipid-lowering, glucose-lowering and blood pressure-lowering drugs; and estimated glomerular filtration rate (eGFR), calculated from serum creatinine, age and sex, assuming that all patients were Caucasian, using the Chronic Kidney Disease Epidemiology Collaboration (CKD-EPI) equation 14. Data on HbA_1c_ were retrieved according to the Mono-S method and were converted to the Diabetes Control and Complications Trial (DCCT) standard through HbA1cDCCT = 0.923 × HbA1cMonoS + 1.345; *R*^2^ = 0.998 15. Data on HbA_1c_ are also reported in International Federation of Clinical Chemistry (IFCC) units (mmol/mol) Patients were categorized as treated with oral glucose-lowering agents alone or with insulin alone or in combination with oral agents.

Data on educational level were obtained by linking the personal identification number to data from the Swedish censuses. Educational level was categorized into two groups: compulsory (9-year comprehensive) school or upper school (including all types of secondary education as upper secondary education or tertiary education), which is the most frequent way of categorizing educational achievement with respect to the educational system in Sweden at that time.

Previous cardiovascular disease was determined by linkage to the Swedish national inpatient registry, using the personal identification number, as a hospitalization for acute myocardial infarction (ICD-10 code I21), heart failure (codes I11.0, I50) or stroke (codes I60,I61,I63.0-I63.5, I63.8-I63.9, I64; corresponding ICD-9 and -8 codes were used for all diagnoses).

### Follow-up and outcomes

The primary endpoint was a composite endpoint of the first non-fatal or fatal event of hospitalization for acute myocardial infarction, heart failure, or stroke (ICD-10 codes as above) or cardiovascular mortality (I00-99). The second endpoints were all-cause mortality and cardiovascular mortality. This endpoint was determined with high validity 16 by linkage to the Swedish national cause-of-death and inpatient registries. Participants were followed from the index date to the first event of an endpoint, emigration or to 31 December 2009.

### Ethical approval

The study, which complied with the declaration of Helsinki, was approved by the Regional Ethical Review Board in Uppsala, Sweden.

### Statistical analysis

The relations of HbA_1c_ to risk of the outcomes were investigated using Cox proportional hazard models, with HbA_1c_ modelled using restricted cubic splines with knots placed at 5th, 35th, 65th and 95th percentiles 17. Covariates in the model were gender (fixed) and age, systolic blood pressure and LDL cholesterol as annually updated means. The time-dependent drug exposures (oral agents or insulin) were entered as a time-varying binary variable, together with an interaction with HbA_1c_ (as restricted cubic spline). Baseline HbA_1c_, age, gender and treatment were required, whereas last observation was carried forward for missing values of annually updated means of HbA_1c_, systolic blood pressure and LDL. Proportional hazard assumptions were assessed by inspecting Schoenfeld residuals.

The value of HbA_1c_ corresponding to lowest risk of an endpoint was searched for in the resulting function. An approximate 95% confidence interval was obtained using 0.025 and 0.975 quintiles of the distribution of optimal HbA_1c_ values estimated by *d*-deletion Jackknife 18 using 500 resamples and letting *d* be half the number of patients.

To assess the robustness of the results, the same analyses were also performed for the subgroup of individuals with no history of cardiovascular disease and further for data sets with imputed data for missing baseline values using multiple imputation technique based on additive regression, bootstrapping and predictive mean matching.

We also investigated relations of HbA_1c_ to risk using deciles of HbA_1c_. Hazard ratios for outcome were analysed by deciles of time, varying HbA_1c_ stratified for time-dependent drug exposures for oral agents and insulin treatment, respectively, where the decile with lowest risk of event was used as the reference level.

The relations of HbA_1c_, primary endpoint and education were further investigated using the Cox proportional hazard model with annually updated HbA_1c_ entered as restricted cubic splines with education level and treatment as fixed and time-varying predictors, respectively. Interaction terms between HbA_1c_ and treatment and education were also entered in the model, which was further adjusted for age. R, version 2.13.1 19 was used for all analyses.

## Results

### Baseline characteristics

During the study period, 19 760 (60%) individuals did not receive insulin and thus remained on oral agents. Table 1 shows the baseline characteristics by treatment category and by education level. Between the educational level categories, the most important difference was that subjects in the compulsory school category were older compared with individuals in the upper school category.

**Table 1:** Baseline characteristics, by (a) treatment category and (b) educational level, in the Retrospective Epidemiological Study to Investigate Outcome and Mortality with Glucose Lowering Drug Treatment in Primary Care (ROSE) study, 1999–2009

(a)			
	Total sample *n* = 32 871	Oral agents *n* = 26 350	Insulin *n* = 6521
Age, years	65.6 (12.1)	65.4 (12.0)	66.8 (12.6)
Female, *n*	14 797 (45%)	11 869 (45%)	2928 (45%)
HbA_1c_, mmol/mol	62 (15)	61 (15)	66 (16)
HbA_1c_,%	7.8 (1.4)	7.7 (1.4)	8.2 (1.5)
Systolic blood pressure, mmHg	146 (18)	146 (18)	145 (19)
Diastolic blood pressure, mmHg	81 (9)	81 (9)	79 (9)
Triglycerides, mm	2.2 (1.6)	2.2 (1.6)	2.0 (1.7)
Total cholesterol, mm	5.3 (1.2)	5.3 (1.1)	5.2 (1.2)
LDL cholesterol, mm	3.2 (1.0)	3.2 (1.0)	3.0 (1.0)
HDL cholesterol, mm	1.5 (0.9)	1.4 (0.9)	1.6 (1.0)
Creatinine, μm	85 (26)	83 (23)	93 (37)
eGFR, ml min^−1^ 1.73 m^−2^	76 (21)	77 (20)	71 (23)
Smoker, *n*	3866 (17%)	3194 (17%)	672 (17%)
Previous cardiovascular disease, *n*	6228 (19%)	4568 (17%)	1660 (26%)
Anti-hypertensive drugs, *n*	16 577 (50%)	13 618 (52%)	2959 (45%)
Lipid-lowering drugs, *n*	8682 (26%)	7339 (28%)	1343 (21%)
Education			
Compulsory school	12 891 (45%)	10 467 (45%)	2424 (47%)
Upper school	15 438 (54%)	12 678 (55%)	2760 (53%)
Oral agents			
Metformin	17 808 (54.2%)	16 758 (63.6%)	1050 (16.1%)
Sulphonylureas	11 335 (34.5%)	10 688 (40.6%)	647 (9.9%)
Meglitinides	528 (1.6%)	498 (1.9%)	30 (0.5%)
Glitazones	306 (0.9%)	293 (1.1%)	13 (0.2%)
Acarbose	254 (0.8%)	234 (0.9%)	20 (0.3%)
DPP-4 inhibitors	37 (0.1%)	35 (0.1%)	2 (0.0%)

(b)			
	Total sample *n* = 28 329	Compulsory school *n* = 12 891	Upper school *n* = 15 438
Age, years	63.5 (11.2)	66.6 (10.4)	60.9 (11.5)
Female, *n*	12 370 (44%)	6103 (47%)	6267 (41%)
HbA_1c_, mmol/mol	62 (15)	63 (15)	62 (15)
HbA_1c_,%	7.8 (1.4)	7.9 (1.4)	7.8 (1.4)
Systolic blood pressure, mmHg	145 (18)	147 (18)	143 (17)
Diastolic blood pressure, mmHg	81 (9)	81 (9)	82 (9)
Triglycerides, mm	2.2 (1.7)	2.2 (1.6)	2.2 (1.6)
Total cholesterol, mm	5.3 (1.1)	5.3 (1.2)	5.3 (1.1)
LDL cholesterol, mm	3.2 (1.0)	3.2 (1.0)	3.2 (1.0)
HDL cholesterol, mm	1.5 (0.9)	1.4 (0.9)	1.5 (0.9)
Creatinine, μm	83 (23)	84 (23)	81 (24)
eGFR, ml min^−1^ 1.73 m^−2^	79 (19)	75 (19)	82 (19)
Smoker, *n*	3717 (18%)	1605 (17%)	2112 (19%)
Previous cardiovascular disease, *n*	4592 (16%)	2501 (19%)	2091 (14%)
Anti-hypertensive drugs, *n*	14 378 (51%)	6811 (53%)	7567 (49%)
Lipid-lowering drugs, *n*	8246 (29%)	3602 (28%)	4644 (30%)
Glucose-lowering agents			
Oral agents, *n*	24 628 (87%)	11 175 (87%)	13 453 (87%)
Metformin	16 568 (58.5%)	7040 (54.6%)	9528 (61.7%)
Sulphonylureas	8869 (31.3%)	4677 (36.3%)	4192 (27.2%)
Meglitinides	494 (1.7%)	198 (1.5%)	296 (1.9%)
Glitazones	290 (1.0%)	105 (0.8%)	185 (1.2%)
Acarbose	222 (0.8%)	104 (0.8%)	118 (0.8%)
DPP-4 inhibitors	37 (0.1%)	11 (0.1%)	26 (0.2%)
Insulin, *n*	5184 (18%)	2424 (19%)	2760 (18%)

Data are means (standard deviations) or numbers of individuals (per cent).

DPP-4, dipeptidyl peptidase-4; eGFR, estimated glomerular filtration rate.

### Primary and secondary outcomes

Median follow-up time for the primary endpoint was 4.8 years. The primary outcome occurred in 8218 (25.0%) cases, total deaths 7814 (23.8%), cardiovascular deaths 5595 (17.0%) and 3575 (10.9%) cases of non-fatal myocardial infarction. The absolute event rates per 1000 person years were 52.7 (51.6–53.8), 33 (32.2–33.9) and 46.2 (45.1–47.2) for primary endpoint, cardiovascular death and all-cause death, respectively.

**FIGURE 1:**
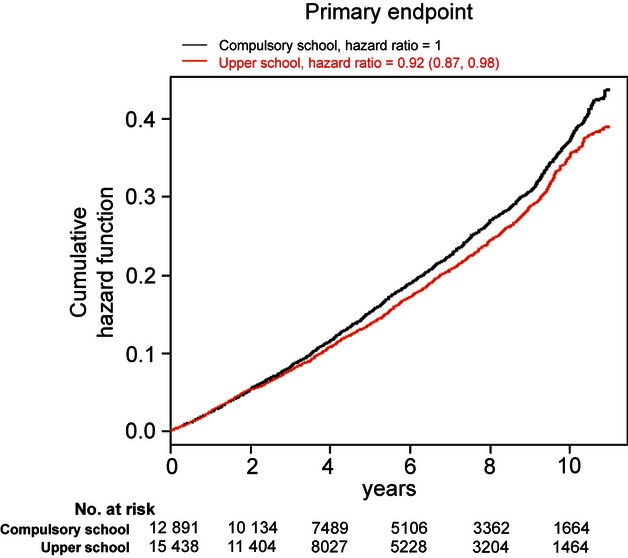
Cox regression model stratified for educational level and adjusted for age. *P*-values from log rank tests.

We further investigated the relation of education level to risk of the primary endpoint, all-cause mortality and cardiovascular mortality. Between the educational levels the location of the density of age differed. Cumulative hazard functions were therefore estimated with Kaplan–Meier stratified for education level as well as with Cox proportional hazard model adjusted for age. Low education defined as compulsory school as highest educational attainment had higher age-adjusted hazard ratio for the primary endpoint compared with subjects with higher education (Fig. 1).

**FIGURE 2:**
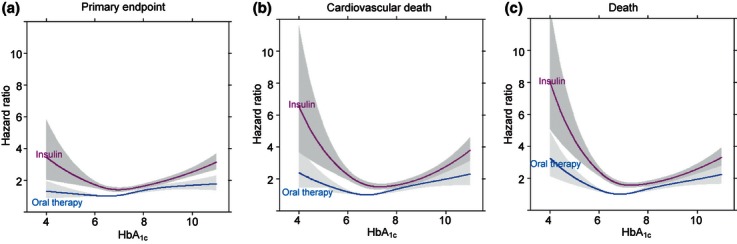
Estimated Cox regression, with HbA_1c_ as annual updated mean as exposure, with age, systolic blood pressure and LDL cholesterol as annual updated mean and gender as covariates. (a) Primary composite endpoint of major cardiovascular events (acute myocardial infarction, heart failure, stroke or cardiovascular death). (b) Cardiovascular death. (c) All-cause mortality.

In multivariable regression spline models adjusted for age, gender, systolic blood pressure and LDL cholesterol, the relations of HbA_1c_ levels to risk of cardiovascular mortality and all-cause mortality were J-shaped, but not to risk of the primary endpoint (Fig. 2). The HbA_1c_ level corresponding to lowest risk of the primary endpoint was 48 mmol/mol (6.5%) [upper confidence limit 50 (6.7)] in subjects on oral agents and 53 mmol/mol (7.0%) [51 (6.8) to 56 (7.3)] for insulin-treated patients. The corresponding figures for all-cause mortality were 51 mmol/mol (6.8%) [51 (6.8) to 53 (7.0)] for oral agents and 56 mmol/mol (7.3%) [54 (7.1) to 61 (7.7)] for insulin treatment; and for cardiovascular death 51 mmol/mol (6.8%) [49 (6.6) to 52 (6.9)] for oral agents and 56 mmol/mol (7.3%) [54 (7.1) to 61 (7.7)] for insulin treatment. All settings were adjusted for age, gender, systolic blood pressure and LDL cholesterol. In sensitivity analyses confined to patients with no history of previous cardiovascular disease, the HbA_1c_ levels at nadir of risk were similar.

Insulin treatment was associated with an increased likelihood of progression to the primary endpoint [1.36 (1.26–1.45)], all-cause mortality [1.51 (1.4–1.62)] and cardiovascular death [1.46 (1.34–1.59)], all *P* < 0.0001 compared with subjects on oral agents, when adjusting for age, gender, systolic blood pressure, LDL and HbA_1c_.The associations between HbA_1c_ and outcomes were further explored. Hazard ratios for outcome are given by deciles of time-varying HbA_1c_ stratified for treatment, confirming an increased risk for cardiovascular mortality and all-cause mortality at both high and low levels of HbA_1c_ in both treatment categories, but not for the primary endpoint and not for subjects in the upper school category (see also Supporting Information, Table S1 and Fig. S1).

### Associations of HbA_1c_ with risk by levels of socio-economic status

The risk for outcomes by deciles of time-varying HbA_1c_ were analysed and stratified for education level, where the decile with lowest risk of event was used as the reference level and adjusted for age, systolic blood pressure and LDL cholesterol and gender (not in Table S1). There was an increased hazard ratio for the primary endpoint [1.3 (1.0–1.6), *P* = 0.0216], all-cause mortality [1.6 (1.3–2.0), *P* < 0.0001] and cardiovascular death [1.6 (1.2–2.1), *P* = 0.0008] at the lowest HbA_1c_ decile for subjects in the low education category. For subjects with higher education, the J-shape curve was evident only for all-cause mortality [1.3 (1.0–1.8), *P* = 0.0266].

**FIGURE 3:**
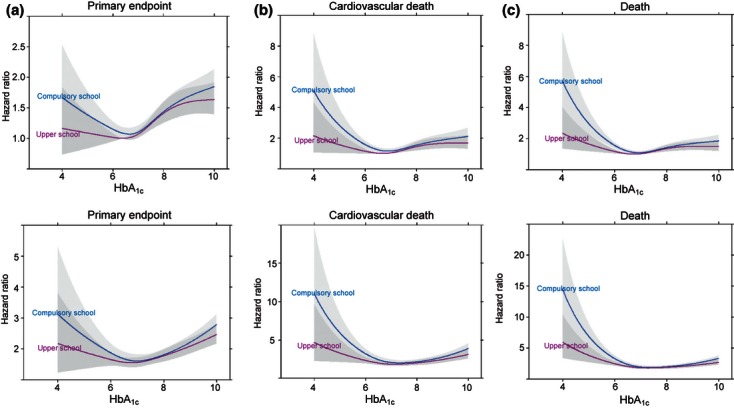
Age-adjusted hazard ratio for (a) primary composite endpoint of major cardiovascular events (acute myocardial infarction, heart failure, stroke or cardiovascular death), (b) cardiovascular death and (c) all-cause mortality. Upper panels are oral agent treatment and lower panels are insulin treatment.

In multivariable regression spline models exploring the association between HbA_1c_ and hazard ratios for primary and secondary endpoints adjusted for age, the J-shaped curve was most evident in patients with low education (Fig. 3). The nadir of risk for the primary endpoint was observed at a slightly higher HbA_1c_ level for subjects with compulsory school as highest educational achievement: 50 mmol/mol (6.7%) [43 (6.1) to 51 (6.8)] vs. 46 mmol/mol (6.4%) [upper confidence limit 50 (6.7)]; in the oral agent treatment category as well as in insulin-treated subjects: 53 mmol/mol (7.0%) [upper confidence limit 57 (7.4)] vs. 51 mmol/mol (6.8%) [upper confidence limit 55 (7.2)], compared with patients with higher educational level.

## Discussion

We found a non-linear relationship between glycaemic control and cardiovascular mortality and all-cause mortality, with elevated risk at both high and low HbA_1c_ levels among patients treated with glucose-lowering medication in primary care. The J-shaped curve was clear in both treatment categories. Furthermore, the J-shaped curve was most marked in patients with low educational level, who also had higher risk for the primary endpoint compared with subjects with higher education. The non-linear associations were confirmed by using both cubic splines and deciles when exploring the relationship between HbA_1c_ and outcome in this retrospective cohort of patients with Type 2 diabetes in primary care.

The observational nature of this study confers a risk of selection bias, and residual or unmeasured confounding may exist, which may limit the validity. Data were missing for smoking status, hence analyses were not adjusted for smoking status; smoking status is known to be socially patterned and may therefore explain some of the observed association between educational level and mortality. Information on diabetes duration was not available, but, using directed acyclic graphs, that was not identified as a crucial confounder. Of note, even if the observed J-shaped relations could partly be explained by confounding, the observed relations were robust across several analyses and subgroups and represent important knowledge as current diabetes and prevention guidelines do not differentiate between reasons for having a particular glucose level.

The main advantage of randomized clinical trials over observational studies is the randomization per se. Meta-analyses of clinical trial study databases, such as investigations of risk in relation to achieved glycaemic control 6,20, have a range of limitations, including heterogeneity of populations and interventions, but retain the advantage of well-matched treatment groups.

The present study also extends those findings to persons who would not match the inclusion criteria of the trials included in those meta-analyses. This is important, as it is well known that patients included in clinical trials differ considerably from those eventually treated with the same drugs in clinical practice 21.

We used low education defined as compulsory school as highest educational attainment as marker for an underprivileged socio-economic group in society. However, other potential markers such as income, type of work, being unemployed or marital status may be equal or more important markers for low socio-economic status. It may also be appropriate to consider the potential roles of co-morbidity, reverse causality and ethnicity (immigrant ethnic groups may have lower educational levels, higher HbA_1c_ and higher mortality than Swedish born groups) in interpreting our findings.

The benefit from intensified glycaemic control nevertheless remains controversial as management recommendations tend to be based on extrapolation from surrogate endpoints. Furthermore, intensified glucose lowering is more difficult to achieve and has greater negative impact on quality of life than lowering cholesterol or blood pressure 22. However, recent trials have questioned the lower-the-better hypothesis for glycaemic control. In the ACCORD trial, high-risk patients with diabetes who were submitted to intensive glycaemic control with an HbA_1c_ target < 6.0% had higher mortality than those with a target of 7.0–7.9% 5. This increased risk was, even if not confirmed, most likely attributable to the increased risk for severe hypoglycaemia in the intensively treated group. Decreased survival in patients achieving low mean percentages of HbA_1c_ might be related to hypoglycaemia—a common complication of intensive blood-glucose control 23. In the Veterans Affairs study 20, more than one episode of severe hypoglycaemia was associated with an 88% higher relative risk for sudden death.

From our study, it is not clear that the J-shaped curve is caused by more frequent hypoglycaemic events in patients with tight glycaemic control. An alternative explanation to the J-shape of the curve may be attributable to reverse causation, i.e. that pre-existing illness explains both the tight glycaemic control and the increased mortality. Furthermore, if pharmacologically induced hypoglycaemia is more dangerous to patients with Type 2 diabetes than previously recognized, this would of course only be relevant to treatment with the potential of inducing hypoglycaemia, i.e. sulphonylureas, meglitinides and insulin. In contrast, metformin, which was the most frequently prescribed oral agent in this study, does not tend to cause hypoglycaemia and the evidence of benefit in terms of reduction in risk of cardiovascular events and mortality is quite strong 24. However, the medication changed as drugs were added, combined or removed continuously during the follow-up time in the study cohort. For this reason, it was not possible to draw conclusions on potential associations between the utilization of a particular class of glucose-lowering agents and outcome, nor was this the scope of the study.

Our results, with a J-shaped association between HbA_1c_ and all-cause mortality and cardiovascular mortality for subjects on oral agents and insulin treatment are in line with a similar recent observational study by Currie *et al*. 9 where data were obtained from routine general practice in the UK. In the present study, the J-curve was not evident for the primary composite endpoint, which primarily consisted of non-fatal (56%) cardiovascular events. This finding is in agreement with the results from the ACCORD trial, where no increased risk for non-fatal cardiovascular events in the intensively treated group was reported 5.

The nadir for the HbA_1c_ level associated with the lowest risk for all-cause mortality and cardiovascular mortality for insulin-treated patients in our study was approximately 7.3%, which is very close to the corresponding nadir of 7.5% in the study from primary care in the UK 9. However, for subjects on oral agents, the nadir for all-cause mortality was lower (6.8%) in our study compared with the study from the UK (7.5%).

While a relationship between socio-economic deprivation and health in the general population is well established, the situation for people with Type 2 diabetes is less clear and a weak or lesser than expected effect of socio-economic deprivation in patients with diabetes is reported 25–27.

We conclude from this observational retrospective cohort study that we were able to confirm some previous results reporting that low and high mean HbA_1c_ values were associated with increased risk for all-cause mortality and cardiovascular mortality in subjects with Type 2 diabetes. Furthermore, the J-shaped curve was most clear in individuals with low educational level. Our results lend support to the recent American Diabetes Association/European Association for the Study of Diabetes position statement that emphasizes the importance of additional factors that should influence HbA_1c_ targets set for individual patients. These include patient attitudes and their expected therapeutic involvement, risk of hypoglycaemia, disease duration, life expectancy, the presence of co-morbidity and patient access to resources and support systems 28.

## Supporting Information

**Table S1.** Hazard ratios for outcome by deciles of time-varying HbA_1c_ stratified for treatment with oral agents and insulin treatment, where the decile with lowest risk of event was used as the reference level within treatment category.

**Figure S1.** Estimated Cox regression, with deciles of HbA_1c_ as annual updated mean exposure, with age, systolic blood pressure and LDL cholesterol as annual updated mean and gender as covariates.

## References

[1] The ADVANCE Collaborative Group (2008). Intensive blood glucose control and vascular outcomes in patients with type 2 diabetes. N Engl J Med.

[2] UK Prospective Diabetes Study (UKPDS) Group (1998). Intensive blood glucose control with sulphonylureas or insulin compared with conventional treatment and risk of complications in patients with type 2 diabetes (UKPDS 33). Lancet.

[3] Bailey CJ, Del Prato S, Eddy D, Zinman B (2005). Earlier intervention in type 2 diabetes: the case for achieving early and sustained glycaemic control. Int J Clin Pract.

[4] Duckworth W, Abraira C, Moritz T, Reda D, Emanuele N, Reaven PD (2009). Glucose control and vascular complications in veterans with type 2 diabetes. N Engl J Med.

[5] The Action to Control Cardiovascular Risk in Diabetes Study Group (2008). Effects of intensive glucose-lowering in type 2 diabetes. N Engl J Med.

[6] Turnbull FM, Abraira C, Anderson RJ, Byington RP, Chalmers JP, Duckworth WC (2009). Intensive glucose control and macrovascular outcomes in type 2 diabetes. Diabetologia.

[7] Boussageon R, Bejan-Angoulvant T, Saadatian-Elahi M, Lafont S, Bergeonneau C, Kassaï B (2011). Effect of intensive glucose lowering treatment on all-cause mortality, cardiovascular death, and microvascular events in type 2 diabetes: meta-analysis of randomised controlled trials. Br Med J.

[8] The Emerging Risk Factors Collaboration (2011). Diabetes mellitus, fasting glucose, and risk of cause-specific death. N Engl J Med.

[9] Currie CJ, Peters JR, Tynan A, Evans M, Heine RJ, Bracco OL (2010). Survival as a function of HbA1c in people with type 2 diabetes: a retrospective cohort study. Lancet.

[10] Huang ES, Liu JY, Moffet HH, John PM, Karter AJ (2011). Glycemic control, complications, and death in older diabetic patients: the diabetes and aging study. Diabetes Care.

[11] Eeg-Olofsson K, Cederholm J, Nilsson PM, Zethelius B, Svensson AM, Gudbjörnsdóttir S (2010). New aspects of HbA1c as a risk factor for cardiovascular diseases in type 2 diabetes: an observational study from the Swedish National Diabetes Register (NDR). J Intern Med.

[12] Robinson N, Lloyd CE, Stevens LK (1998). Social deprivation and mortality in adults with diabetes mellitus. Diabet Med.

[13] Martinell M, Stålhammar J, Hallqvist J (2012). Automated data extraction – a feasible way to construct patient registers of primary care utilization. Ups J Med Sci.

[14] Levey AS, Stevens LA, Schmid CH, Zhang YL, Castro AF, Feldman HI (2009). A new equation to estimate glomerular filtration rate. Ann Intern Med.

[15] Hoelzel W, Weykamp C, Jeppsson JO, Miedema K, Barr JR, Goodall I (2004). IFCC reference system for measurement of hemoglobin A1c in human blood and the national standardization schemes in the United States, Japan, and Sweden: a method–comparison study. Clin Chem.

[16] Ludvigsson JF, Andersson E, Ekbom A, Feychting M, Kim JL, Reuterwall C (2011). External review and validation of the Swedish national inpatient register. BMC Public Health.

[17] Harrel FE (2001). Regression Modelling Strategies. With Applications to Linear Models, Logistic Regression and Survival Analysis.

[18] Shao J, Tu D (1995). The Jackknife and Bootstrap.

[19] R Development Core Team (2011). R: A Language and Environment for Statistical Computing.

[20] Ray KK, Seshasai SR, Wijesuriya S, J Sivakumaran R, Nethercott S, Preiss D (2009). Effect of intensive control of glucose on cardiovascular outcomes and death in patients with diabetes mellitus: a meta-analysis of randomized controlled trials. Lancet.

[21] Björklund E, Lindahl B, Stenestrand U, Swahn E, Dellborg M, Pehrsson K (2004). Outcome of ST-elevation myocardial infarction treated with thrombolysis in the unselected population is vastly different from samples of eligible patients in a large-scale clinical trial. Am Heart J.

[22] Huang ES, Brown SES, Ewigman BG, Foley EC, Meltzer DO (2007). Patient perceptions of quality of life with diabetes-related complications and treatments. Diabetes Care.

[23] Miller CD, Phillips LS, Ziemer DC, Gallina DL, Cook CB, El-Kebbi IM (2001). Hypoglycemia in patients with type 2 diabetes mellitus. Arch Intern Med.

[24] Holman RR, Paul SK, Bethel MA, Matthews DR, Neil HA (2008). 10-year follow-up of intensive glucose control in type 2 diabetes. N Engl J Med.

[25] Koskinen SV, Martelin TP, Valkonen T (1996). Socio-economic differences in mortality among diabetic people in Finland: 5-year follow-up. Br Med J.

[26] Gnavi R, Petrelli A, Spadea MDT, Carta Q, Costa G (2004). Mortality and education level among diabetic and non-diabetic population in the Turin Longitudinal Study: a 9-year follow-up. Int. J. Epidemiol.

[27] O'Connor R, Houghton F, Saunders J, Dobbs F (2006). Diabetes mellitus in Irish general practice: levels of care as reflected by HbA1c values. Eur J Gen Prac.

[28] Inzucchi SE, Bergenstal RM, Buse JB, Inzucchi SE, Bergenstal RM, Buse JB (2012). Management of hyperglycaemia in type 2 diabetes: a patient-centered approach. Position statement of the American Diabetes Association (ADA) and the European Association for the Study of Diabetes (EASD). Diabetologia.

