# Supplementary figures and images for: Associations of HbA1c and educational level with risk of cardiovascular events in 32 871 drug-treated patients with Type 2 diabetes: a cohort study in primary care
Source: Diabet Med. 2013 Mar 13;30(5):e170–7. doi: 10.1111/dme.12145 (PMC3654570; doi:10.1111/dme.12145)

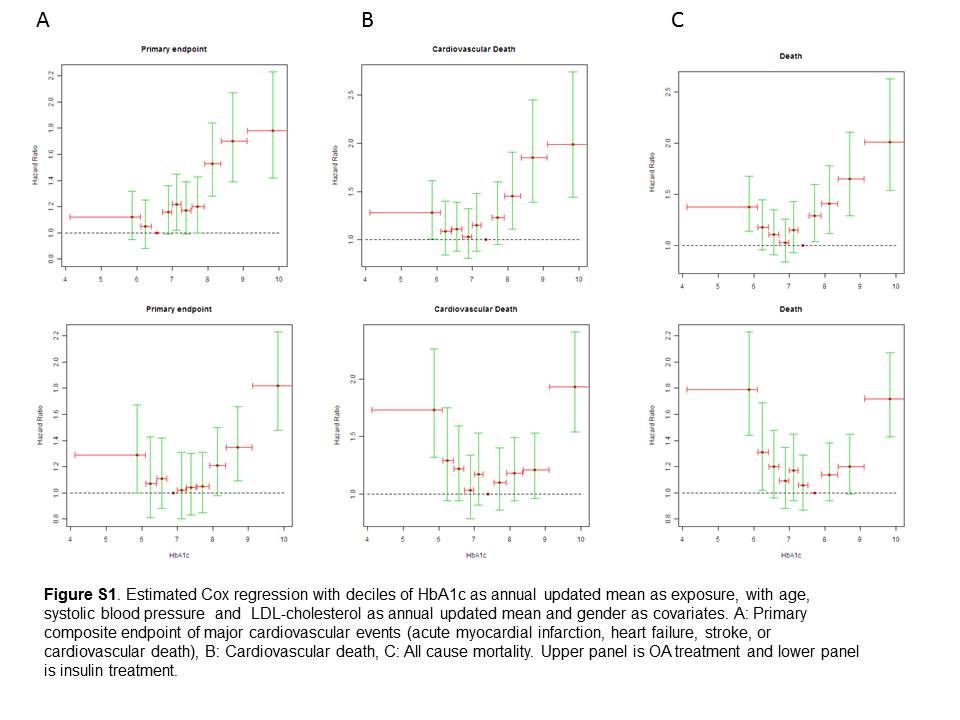

Supplement: Supplementary file 1 [file dme0030-e170-SD1.jpg]
