# Supplementary material for: Associations of HbA1c and educational level with risk of cardiovascular events in 32 871 drug-treated patients with Type 2 diabetes: a cohort study in primary care
Source: Diabet Med. 2013 Mar 13;30(5):e170–7. doi: 10.1111/dme.12145 (PMC3654570; doi:10.1111/dme.12145)
Supplement: Supplementary file 2 [file dme0030-e170-SD2.docx]

**Table S1.** HR for outcome by deciles (D) of time varying HbA1c stratified for treatment with oral agents and insulin treatment, where the decile with lowest risk of event was used as the reference level within treatment categoty. Adjusted for age, systolic blood pressure and LDL-cholesterol as annual updated mean and gender as covariates. The ROSE study, 1999-2009.

**Oral agents Insulin Insulin vs. oral agents**

Events/person years HR P Events/person years HR P HR P

***PRIMARY ENDPOINT (All)***

D1 267/8721 1.12 (0.95-1.32) 0.1644 104/1896 1.29 (1-1.67) 0.0532 1.64 (1.3-2.07) <0.0001

D2 215/7358 1.05 (0.88-1.25) 0.6107 74/15466 1.07 (0.81-1.43) 0.6268 1.45 (1.11-1.91) 0.0067

D3 309/10646 **1**  136/2681 1.11 (0.88-1.42) 0.3724 1.63 (1.32-2.01) <0.0001

D4 290/8680 1.16 (0.99-1.36) 0.0754 133/2869 **1**  1.32 (1.07-1.63) 0.0104

D5 216/6161 1.22 (1.02-1.45) 0.0276 121/2601 1.02 (0.8-1.31) 0.8641 1.22 (0.96-1.54) 0.0981

D6 245/7359 1.17 (0.99-1.39) 0.0606 184/4057 1.04 (0.83-1.3) 0.7094 1.27 (1.04-1.56) 0.0174

D7 191/5748 1.2 (1-1.43) 0.0522 204/4486 1.05 (0.85-1.31) 0.6451 1.34 (1.09-1.65) 0.0055

D8 188/4601 1.53 (1.28-1.84) <0.0001 249/4860 1.21 (0.98-1.5) 0.0712 1.18 (0.97-1.44) 0.0992

D9 142/3488 1.7 (1.39-2.07) <0.0001 273/5024 1.35 (1.09-1.66) 0.0049 1.16 (0.94-1.43) 0.1669

D10 106/2940 1.78 (1.42-2.23) <0.0001 306/4983 1.82 (1.48-2.23) <0.0001 1.59 (1.26-2.01) 0.0001

***DEATH (All)***

D1 268/9241 1.38 (1.14-1.68) 0.0011 139/2076 1.79 (1.44-2.23) <0.0001 1.93 (1.56-2.38) <0.0001

D2 198/7784 1.18 (0.96-1.45) 0.1134 89/1701 1.31 (1.02-1.69) 0.0344 1.63 (1.26-2.11) 0.0002

D3 273/11231 1.11 (0.91-1.35) 0.2912 146/2997 1.2 (0.96-1.48) 0.1022 1.66 (1.35-2.04) <0.0001

D4 207/9218 1.03 (0.84-1.26) 0.8094 146/3238 1.09 (0.88-1.35) 0.4495 1.69 (1.35-2.1) <0.0001

D5 161/6531 1.15 (0.93-1.43) 0.2075 142/2949 1.17 (0.94-1.45) 0,1543 1.65 (1.3-2.1) <0.0001

D6 166/7878 **1** 191/4667 1.06 (0.87-1.29) 0.5689 1.52 (1.22-1.89) 0.0002

D7 160/6165 1.29 (1.04-1.6) 0.0219 193/5049 **1** 1.23 (0.99-1.53) 0.0651

D8 131/4942 1.41 (1.12-1.78) 0.0032 235/5523 1.14 (0.94-1.38) 0.1718 1.37 (1.09-1.71) 0.0060

D9 103/3728 1.65 (1.29-2.11) 0.0001 245/5775 1.2 (0.99-1.45) 0.0614 1.13 (0.89-1.44) 0.2988

D10 80/3056 2.01 (1.54-2.63) <0.0001 285/5646 1.72 (1.43-2.07) <0.0001 1.39 (1.07-1.8) 0.0133

***CARDIOVASCULAR DEATH (All)***

D1 174/9241 1.28 (1.01-1.61) 0.0419 91/2076 1.73 (1.32-2.26) 0.0001 1.9 (1.46-2.46) <0.0001

D2 128/7784 1.09 (0.84-1.4) 0.5198 60/1701 1.29 (0.94-1.75) 0.1116 1.67 (1.22-2.29) 0.0015

D3 192/11231 1.11 (0.88-1.39) 0.383 103/2997 1.22 (0.94-1.59) 0.1299 1.63 (1.27-2.08) 0.0001

D4 147/9218 1.03 (0.81-1.32) 0.7874 95/3238 1.03 (0.78-1.34) 0.8548 1.54 (1.18-2.01) 0.0017

D5 113/6531 1.15 (0.88-1.48) 0.3039 99/2949 1.17 (0.9-1.53) 0.2453 1.61 (1.21-2.15) 0.0012

D6 117/7878 **1**  123/4667 **1**  1.41 (1.09-1.84) 0.0098

D7 108/6165 1.23 (0.95-1.6) 0.1138 145/5049 1.1 (0.86-1.4) 0.4442 1.35 (1.04-1.75) 0.0240

D8 95/4942 1.45 (1.11-1.91) 0.0068 167/5523 1.18 (0.94-1.49) 0.1588 1.28 (0.99-1.67) 0.0640

D9 81/3728 1.85 (1.39-2.45) <0.0001 169/5775 1.21 (0.96-1.53) 0.105 0.97 (0.74-1.28) 0.8495

D10 55/3056 1.99 (1.44-2.74) <0.0001 214/5646 1.93 (1.54-2.41) <0.0001 1.49 (1.1-2.03) 0.0108

***PRIMARY ENDPOINT (Compulsory school)***

D1 118/3613 1.32 (1.02-1.71) 0.0317 49/780 1.34 (0.92-1.94) 0.1262 1.88 (1.33-2.65) 0.0003

D2 94/3109 1.2 (0.91-1.57) 0.1955 24/628 0.77 (0.48-1.23) 0.2726 1.17 (0.74-1.85) 0.5054

D3 116/4531 **1**  61/1197 1.05 (0.74-1.49) 0.7731 1.8 (1.3-2.49) 0.0004

D4 121/3766 1.27 (0.98-1.64) 0.0674 65/1320 **1**  1.47 (1.07-2.01) 0.0171

D5 98/2856 1.37 (1.05-1.79) 0.0220 50/1188 0.89 (0.62-1.29) 0.5422 1.05 (0.73-1.51) 0.7926

D6 103/3428 1.22 (0.93-1.59) 0.1431 90/1883 1 (0.73-1.38) 0.9991 1.42 (1.05-1.92) 0.0211

D7 85/2636 1.33 (1.01-1.77) 0.0443 85/2155 0.84 (0.61-1.16) 0.2978 1.25 (0.91-1.71) 0.1639

D8 86/2154 1.72 (1.3-2.28) 0.0001 104/2308 0.99 (0.72-1.34) 0.9244 1.04 (0.77-1.41) 0.7928

D9 69/1619 2.03 (1.5-2.73) <0.0001 125/2316 1.21 (0.9-1.64) 0.2068 1.05 (0.78-1.43) 0.7422

D10 43/1208 1.91 (1.34-2.72) 0.0004 148/2139 1.75 (1.31-2.35) 0.0002 1.85 (1.3-2.64) 0.0007

***DEATH (Compulsory school)***

D1 117/3871 1.69 (1.24-2.3) 0.0009 62/872 2.6 (1.84-3.67) <0.0001 2.06 (1.5-2.82) <0.0001

D2 88/3313 1.45 (1.05-2.01) 0.0247 28/699 1.39 (0.89-2.15) 0.1473 1.28 (0.83-1.98) 0.2700

D3 93/4824 1.06 (0.77-1.46) 0.7191 60/1341 1.55 (1.1-2.2) 0.0129 1.83 (1.3-2.56) 0.0005

D4 65/4039 0.9 (0.64-1.28) 0.5668 69/1511 1.52 (1.09-2.13) 0.0144 2.32 (1.63-3.31) <0.0001

D5 71/3027 1.36 (0.97-1.92) 0.0748 54/1359 1.39 (0.97-1.99) 0.0715 1.31 (0.9-1.91) 0.1630

D6 62/3654 **1** 80/2193 1.28 (0.92-1.76) 0.1381 1.45 (1.02-2.06) 0.0373

D7 58/2817 1.25 (0.87-1.78) 0.2278 68/2459 **1** 1.11 (0.77-1.6) 0.5784

D8 56/2329 1.54 (1.07-2.21) 0.0190 81/2656 1.15 (0.83-1.59) 0.3936 1.13 (0.79-1.61) 0.5046

D9 41/1738 1.7 (1.15-2.53) 0.0083 102/2703 1.46 (1.07-1.99) 0.0156 1.15 (0.79-1.67) 0.4651

D10 29/1256 2.04 (1.31-3.17) 0.0016 135/2492 2.38 (1.77-3.18) <0.0001 1.65 (1.09-2.49) 0.0183

***CARDIOVASCULAR DEATH (Compulsory school)***

D1 74/3871 1.51 (1.04-2.19) 0.0320 44/872 2.27 (1.51-3.4) 0.0001 2.29 (1.56-3.37) <0.0001

D2 58/3313 1.35 (0.91-1.99) 0.1369 17/699 1.03 (0.59-1.78) 0.9241 1.15 (0.66-2.01) 0.6165

D3 59/4824 0.95 (0.64-1.4) 0.7851 42/1341 1.33 (0.88-2) 0.1731 1.97 (1.31-2.97) 0.0012

D4 48/4039 0.94 (0.62-1.42) 0.7694 51/1511 1.37 (0.93-2.01) 0.1159 2.33 (1.54-3.51) 0.0001

D5 48/3027 1.3 (0.87-1.96) 0.2034 33/1359 1.05 (0.67-1.62) 0.8429 1.15 (0.72-1.84) 0.5674

D6 44/3654 **1**  51/2193 **1**  1.29 (0.84-1.97) 0.2434

D7 38/2817 1.15 (0.75-1.78) 0.5175 50/2459 0.91 (0.61-1.34) 0.6216 1.25 (0.8-1.94) 0.3328

D8 41/2329 1.6 (1.05-2.46) 0.0297 56/2656 0.98 (0.67-1.43) 0.9202 1.02 (0.67-1.55) 0.9375

D9 32/1738 1.9 (1.2-3) 0.0058 76/2703 1.35 (0.94v1.92) 0.1018 1.04 (0.68-1.59) 0.8678

D10 20/1256 2.02 (1.19-3.43) 0.0096 102/2492 2.24 (1.6-3.14) <0.0001 1.86 (1.14-3.05) 0.0134

***PRIMARY ENDPOINT (Upper school)***

D1 91/4803 1.06 (0.81-1.41) 0.6637 34/181055 1.21 (0.77-1.92) 0.4024 1.44 (0.96-2.17) 0.0790

D2 83/4035 1.11 (0.83-1.47) 0.4880 28/831 1.28 (0.79-2.07) 0.3163 1.43 (0.92-2.24) 0.1133

D3 109/5721 **1**  45/1369 1.19 (0.78-1.82) 0.4208 1.58 (1.1-2.27) 0.0130

D4 110/4648 1.22 (0.93-1.59) 0.1468 41/1446 **1**  1.21 (0.83-1.76) 0.3114

D5 67/3081 1.15 (0.85-1.56) 0.3712 44/1307 1.19 (0.78-1.82) 0.4211 1.4 (0.94-2.09) 0.0948

D6 89/3650 1.31 (0.99-1.73) 0.0625 67/2038 1.24 (0.84-1.83) 0.2745 1.2 (0.86-1.67) 0.2760

D7 66/2816 1.29 (0.95-1.75) 0.1071 69/2147 1.25 (0.85-1.84) 0.2580 1.16 (0.82-1.66) 0.4012

D8 66/2211 1.7 (1.25-2.32) 0.0007 100/2321 1.7 (1.18-2.45) 0.0041 1.39 (1-1.92) 0.0484

D9 38/1674 1.46 (1-2.11) 0.0473 96/2447 1.68 (1.17-2.43) 0.0053 1.64 (1.1-2.45) 0.0147

D10 39/1554 1.83 (1.26-2.66) 0.0014 106/2608 2.03 (1.42-2.93) 0.0001 1.47 (0.99-2.19) 0.0566

***DEATH (Upper school)***

D1 68/5023 1.02 (0.71-1.46) 0.9130 46/1131 1.61 (1.09-2.37) 0.0157 2.32 (1.57-3.41) <0.0001

D2 51/4207 0.9 (0.61-1.32) 0.5911 30/894 1.31 (0.84-2.04) 0.2296 2.13 (1.33-3.41) 0.0016

D3 67/5954 0.82 (0.57-1.18) 0.2798 41/1519 1.01 (0.68-1.5) 0.9658 1.89 (1.26-2.83) 0.0020

D4 74/4860 1.06 (0.75-1.51) 0.7305 41/1598 0.93 (0.62-1.38) 0.7119 1.48 (0.99-2.19) 0.0543

D5 40/3234 0.89 (0.59-1.35) 0.5931 41/1460 1.01 (0.68-1.5) 0.9676 1.8 (1.13-2.85) 0.0129

D6 53/3901 **1** 62/2299 1.05 (0.74-1.5) 0.7810 1.57 (1.08-2.29) 0.0185

D7 46/3006 1.22 (0.82-1.81) 0.3291 59/2360 **1** 1.17 (0.78-1.75) 0.4411

D8 27/2344 0.96 (0.6-1.53) 0.8700 83/2595 1.29 (0.92-1.8) 0.1348 2.28 (1.45-3.57) 0.0003

D9 22/1757 1.23 (0.75-2.03) 0.4134 63/2737 1.04 (0.73-1.49) 0.8160 1.39 (0.84-2.29) 0.2002

D10 22/1603 1.73 (1.05-2.86) 0.0317 84/2839 1.58 (1.13-2.21) 0.0073 1.42 (0.87-2.31) 0.1623

***CARDIOVASCULAR DEATH (Upper school)***

D1 38/5023 0.96 (0.6-1.53) 0.8516 26/1131 1.45 (0.88, 2.41) 0.1442 2.23 (1.32-3.75) 0.0025

D2 27/4207 0.8 (0.48-1.33) 0.3830 17/894 1.17 (0.66, 2.08) 0.5906 2.15 (1.14-4.05) 0.0177

D3 47/5954 0.96 (0.61-1.5) 0.8500 24/1519 0.93 (0.56, 1.55) 0.7808 1.44 (0.87-2.39) 0.1612

D4 43/4860 1.02 (0.65-1.61) 0.9302 17/1598 0.6 (0.34, 1.06) 0.0778 1.06 (0.59-1.9) 0.8425

D5 24/3234 0.89 (0.52-1.51) 0.6685 28/1460 1.17 (0.9-1.53) 0.2453 1.93 (1.08-3.45) 0.0260

D6 32/3901 **1**  37/2299 **1**  1.57 (0.97-2.56) 0.0670

D7 32/3006 1.41 (0.86-2.29) 0.1737 42/2360 1.13 (0.72, 1.76) 0.5932 1.13 (0.7-1.81) 0.6228

D8 16/2344 0.94 (0.52-1.72) 0.8420 60/2595 1.47 (0.98, 2.22) 0.0652 2.62 (1.48-4.62) 0.0009

D9 17/1757 1.6 (0.89-2.89) 0.1176 38/2737 1.02 (0.65, 1.61) 0.9297 1.09 (0.6-1.97) 0.7751

D10 13/1603 1.76 (0.92-3.36) 0.0893 61/2839 1.91 (1.27, 2.88) 0.0020 1.74 (0.93-3.24 0.0814
